# Pharmacokinetic-pharmacodynamic correlations in the development of ginger extract as an anticancer agent

**DOI:** 10.1038/s41598-018-21125-2

**Published:** 2018-02-14

**Authors:** Rao Mukkavilli, Chunhua Yang, Reenu Singh Tanwar, Roopali Saxena, Sushma R. Gundala, Yingyi Zhang, Ahmed Ghareeb, Stephan D. Floyd, Subrahmanyam Vangala, Wei-Wen Kuo, Padmashree C. G. Rida, Ritu Aneja

**Affiliations:** 1 Department of Biology, Georgia State University, Atlanta, GA-30303 USA; 2 ReaGene Biosciences Private Limited, Bengaluru, India; 3 Department of Biological Science and Technology, College of Biopharmaceutical and Food Sciences, China Medical University, Taichung, Taiwan, ROC

## Abstract

Anticancer efficacy of ginger phenolics (GPs) has been demonstrated in various *in vitro* assays and xenograft mouse models. However, only sub-therapeutic plasma concentrations of GPs were detected in human and mouse pharmacokinetic (PK) studies. Intriguingly, a significant portion of GPs occurred as phase II metabolites (mainly glucuronide conjugates) in plasma. To evaluate the disposition of GPs and understand the real players responsible for efficacy, we performed a PK and tissue distribution study in mice. Plasma exposure of GPs was similar on day 1 and 7, suggesting no induction or inhibition of clearance pathways. Both free and conjugated GPs accumulated in all tissues including tumors. While non-cytotoxicity of 6-ginerol glucuronide precluded the role of conjugated GPs in cell death, the free forms were cytotoxic against prostate cancer cells. The efficacy of ginger was best explained by the reconversion of conjugated GPs to free forms by β-glucuronidase, which is over-expressed in the tumor tissue. This previously unrecognized two-step process suggests an instantaneous conversion of ingested free GPs into conjugated forms, followed by their subsequent absorption into systemic circulation and reconversion into free forms. This proposed model uncovers the mechanistic underpinnings of ginger’s anticancer activity despite sub-therapeutic levels of free GPs in the plasma.

## Introduction

Recently, there has been a resurgence of whole food-based therapeutics categorized as Natural Medicines (NMs) in prevention and treatment of chronic diseases like cancer^1,2^. However, their clinical development has suffered perhaps due to sub-therapeutic concentrations of constituent phytochemicals in the plasma. Ginger is an example. An almost instantaneous conversion of the ingested free ginger phenolics (GPs) into their pharmacologically-inactive phase II metabolites (glucuronides, sulfates, etc.) in the plasma has perpetuated a diminished interest in their development. However, this highlights a knowledge gap as to how constituent phytochemicals contribute physiologically to the observed pharmacological effects of NMs. Undoubtedly, NMs are a complex concoction of chemically-diverse phytochemicals containing one to several bioactive compounds, which mostly are plant secondary metabolites. Recently, a unique approach called ‘*reverse pharmacokinetics’* has helped to draw possible reasons underlying the discordance between pharmacokinetics (PK) and pharmacodynamics (PD) of NMs^3^. In this study, we examined how the free (active) and conjugated (inactive) species interconvert on the physiological time scale to ultimately pinpoint the bioactive species responsible for the PD effect. For almost a decade now, our laboratory has been studying the pharmacology of ginger root extract. Thus, ginger extract was our obvious choice as the probe NM to investigate the pharmacokinetic-pharmacodynamic (PK-PD) relationships between free and conjugated forms of GPs to precisely define the role of Phase II metabolites in the anticancer activity of ginger extract.

Ginger (*Zingiber officinale*), a widely consumed spice worldwide, is an excellent source of phenolic compounds including gingerols, shogaols, paradols, gingerdiones etc.^4–6^. Literature has extensively demonstrated that 6-gingerol (6G), 8-gingerol (8G), 10-gingerol (10G) and 6-shogaol (6S) collectively referred to as ginger phenolics (GPs, Supplementary Figure 1), are the most abundant bioactive constituents of whole ginger extract, and exhibit anti-proliferative, anti-inflammatory, anti-oxidative and anti-tumor properties^4,7–12^. We have published the tumor growth-inhibitory efficacy of ginger extract (GE) in prostate cancer models as well as reported the *in vitro* and *in vivo* synergistic interrelationships among various ginger constituents when administered in their original matrix^13,14^. Furthermore, we have shown that GPs undergo enterohepatic recirculation and their glucuronide conjugates circulate in the system^15^. We also demonstrated that ginger extract (GE, 250 mg/kg) upon daily oral administration for 28 days in mice showed 68% tumor growth inhibition compared to controls. A quasi-mixture of 6G, 8G, 10G and 6S (equivalent to what is present in GE) showed 28% tumor inhibition and GE devoid of these components showed 35% tumor inhibition. These experiments confirmed the presence of other active constituents in GE which have anti-cancer potential and that whole ginger is better than its individual constituents. In a follow up study, we dosed GE with ketoconazole in mice, which confirmed that phase II metabolism is critical for clearance of GE phenolics than Phase I, as concentration time profile of GPs was more or less similar with or without concomitant ketoconazole administration^16^. However, studies by Zick *et al*. revealed that sufficient (or therapeutic) plasma concentrations of these bioactive GPs are not achieved in humans despite a high dose of 2 g per day. Instead of free gingerols, glucuronide and sulfate conjugates of 6G, 8G, 10G and 6S were observed in plasma^17^. There is a widely-held notion that phase II conjugates are water-soluble, eliminated from the system quickly and most of them are pharmacologically inactive. There are no available reports that describe whether these conjugates contribute to or modulate the anticancer efficacy of the whole ginger extract. The disconnect between the observed efficacy and sub-therapeutic plasma concentrations of free GPs generate compelling grounds to further investigate the fate of the GPs and their metabolites *in vivo*.

To gain insights into the interrelationships between free and conjugated forms of GPs, we conducted pharmacokinetic study in healthy mice and tissue distribution in mice bearing human xenografts. In addition, *in-vitro* cytotoxicity potential was assessed for GPs compared to 6G-glucuronide. Our results clearly demonstrate the accumulation of free and conjugated GPs in various tissues including tumors. The selective over-expression of β-glucuronidase (β-gd) in cancer cells compared to normal cells facilitates the conversion of conjugated glucuronides to free forms in tumor tissue. *In vitro* studies confirmed that the free forms are more cytotoxic compared to the glucuronide conjugates.

## Results

### Pharmacokinetics of GPs following daily administration of GE for seven days

**Figure 1:**
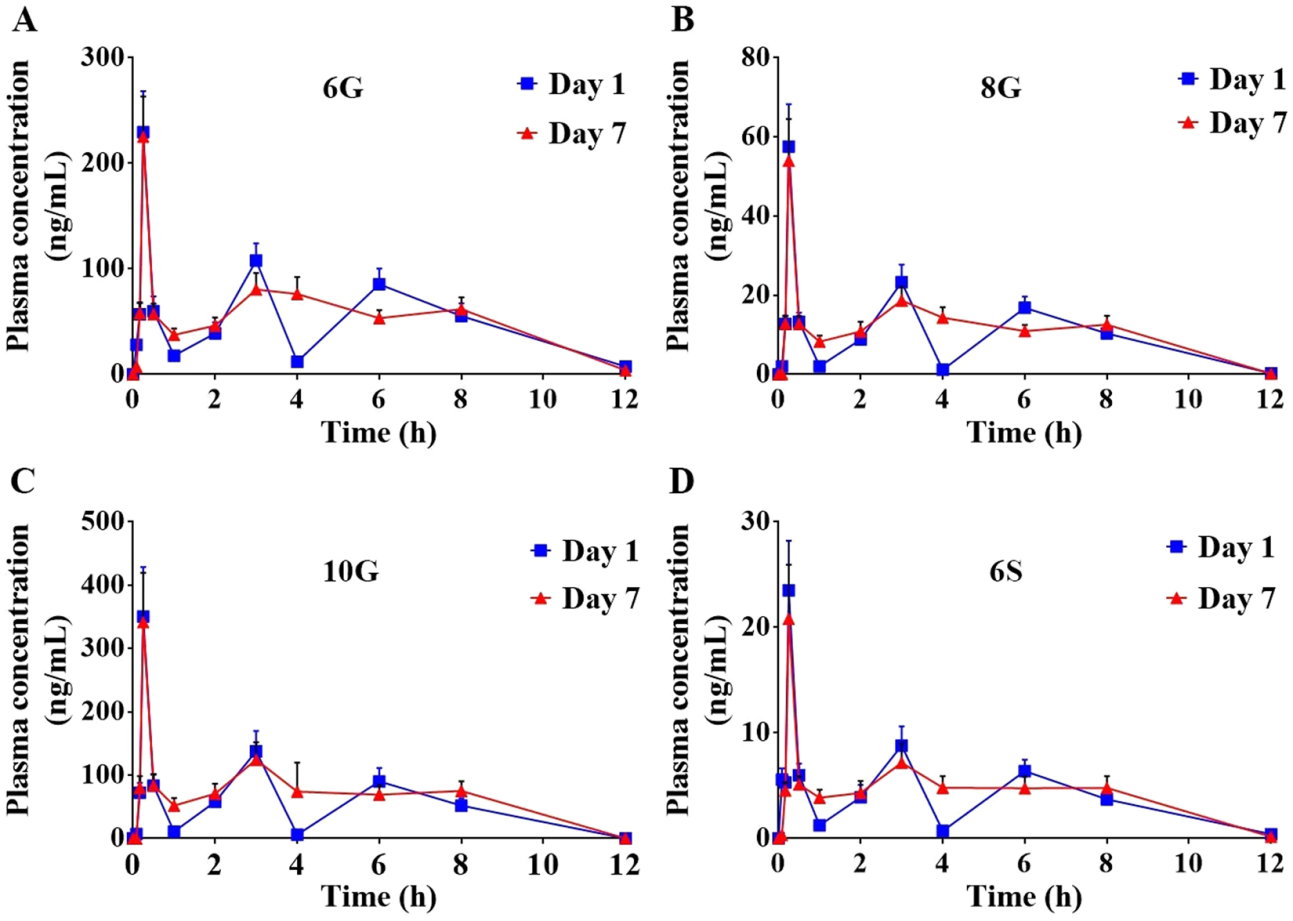
Comparison of plasma kinetics on day 1 vs. day 7 of Ginger phenolics following daily dose administration of GE at 250 mg/kg. The plasma concentrations of (**A**) 6G, (**B**) 8G, (**C**) 10G and (**D**) 6S were quantitated in samples collected at various time points (0, 0.08, 0.16, 0.25, 0.5, 1, 2, 3, 4, 6, 8 and 12 h) on day 1 vs day 7 following oral administration of GE. Values and error bars shown in the figure represent mean and standard deviation (SD), respectively.

We compared the plasma concentration-time profiles of 6G, 8G, 10G and 6S on day 1 and day 7 following repeated daily oral administration of GE. Exposure (AUC_last_) of 6G, 8G, 10G and 6S was similar on day 7 compared to day 1 (Table 1, Fig. 1), suggesting no induction or inhibition of their clearance pathways. Furthermore, the presence of multiple peaks was evident on day 1 for all GPs, confirming that 6G, 8G, 10G, and 6S were undergoing enterohepatic recirculation. While prominent peaks were observed on day 1 concentration-time profile, peaks were less pronounced on day 7.

**Table 1:** Comparison of pharmacokinetic parameters of GE phenolics on day 1 vs day 7 following daily oral administration of GE in C57BL/6 J mice (dose: 250 mg/kg, n = 3 per time point, mean ± SE).

PK Parameters	6G		8G		10G		6S	
	Day 1	Day 7	Day 1	Day 7	Day 1	Day 7	Day 1	Day 7
T_max_ (h)	0.25	0.25	0.25	0.25	0.25	0.25	0.25	0.25
C_max_ (ng/mL)	229.66 ± 67.32	225.37 ± 65.94	57.61 ± 18.53	54.01 ± 18.22	350.74 ± 134.57	341.87 ± 134.09	23.53 ± 8.17	20.83 ± 8.86
C_max_ Ratio	0.98		0.94		0.97		0.89	
AUC_last_ (ng.h/mL)	614.97 ± 195.92	641.46 ± 195.10	116.84 ± 34.38	132.36 ± 17.28	1244.40 ± 97.96	1596.71 ± 424.31	47.39 ± 15.29	51.88 ± 20.13
AUC_last_ Ratio	1.04		1.13		1.28		1.09	

T_max_: time to reach peak plasma concentration; C_max_: peak plasma concentration; AUC_last_: area under the curve; SE: standard error; ratio: day 7/day 1; The exposure (C_max_ and AUC_last_) values of 6G, 8G, 10G and 6S in plasma samples obtained following oral administration of GE were compared using independent sample t-test and were found to be non-significant. The C_max_ and AUC_last_ ratios of Day 7 to Day 1 are presented in the table.

The serving size of ginger in humans varies from 250 mg to 4.8 g per day. Ginger extract is generally regarded as safe (GRAS) ingredient in accordance with US FDA^18^. Studies report that GE contains 5% of active GPs, i.e. 12.5 mg in 250 mg/kg dose, and has been a dose level of choice for therapeutic studies^15,17^. Thus, we used GE with similar concentration for all our studies with 1 mg containing 30.03 µg of 6G, 6.86 µg of 8G, 7.77 µg of 10G, 5.96 µg of 6S in the ratio of 5:1:1:1. While the plasma exposure (AUC_last_) of 10G (1244.40 ng.h/mL) was twice that of 6G (614.97 ng.h/mL), plasma exposure of 8G (116.84 ng.h/mL) was 5 times lower than 6G. Notably, the plasma exposure of 6S was lowest at (AUC_last_) 47.39 ng.h/mL, indicating its low bioavailability compared to other GPs.

### Tissue distribution of GPs

**Figure 2:**
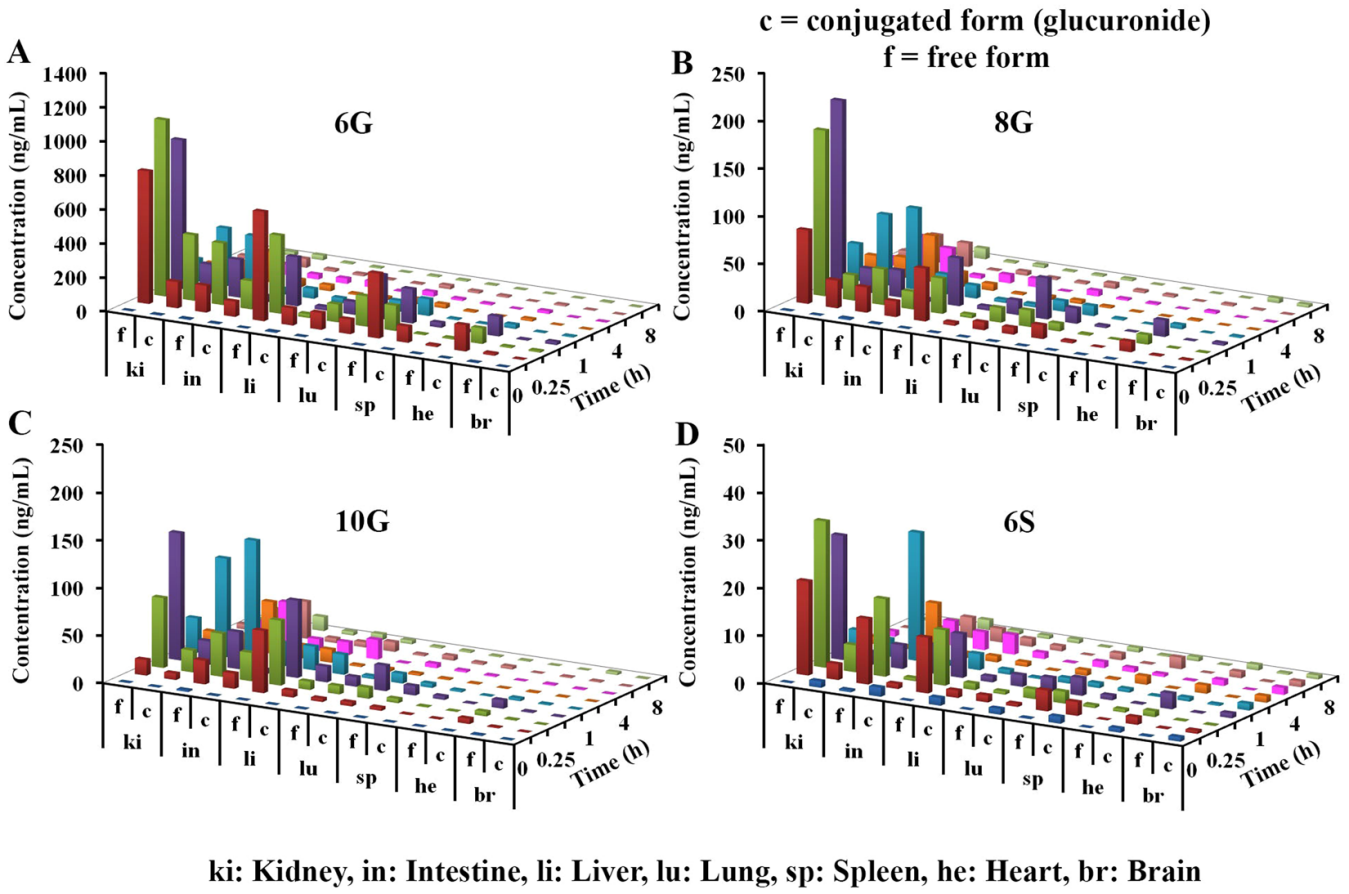
Tissue distribution of GPs following daily dose administration of GE for 7 days. Concentrations of GPs in untreated and β-glucuronidase treated samples obtained from the intestine, liver, spleen, heart, brain, lung and kidney were compared at different time points (n = 3). Three-dimensional representation of graphs in Microsoft Excel does not permit SD inclusion and hence concentration-time data with SD is presented in Supplementary Tables 2–8.

Having quantitated the free and conjugated GPs in the plasma, we next determined their distribution across various tissues in tumor-bearing mice. Stomach, intestine, liver, spleen, heart, brain, lung, kidney, and tumor were collected on day 7 following repeated oral administration and were processed for quantitative analysis. Since we have earlier observed^15^ that majority of free GPs are converted to their respective glucuronides, the tissue samples were treated with β-glucuronidase to determine the concentration of conjugated forms of 6G, 8G, 10G, and 6S. Essentially, the concentration of glucuronide conjugates was calculated by subtracting the free analyte concentration from the total concentration obtained from β-gd treated samples (Supplementary Tables 1–9). Our results indicated the presence of glucuronide conjugates of GPs in all harvested tissues in addition to their free forms (Fig. 2).

**Figure 3:**
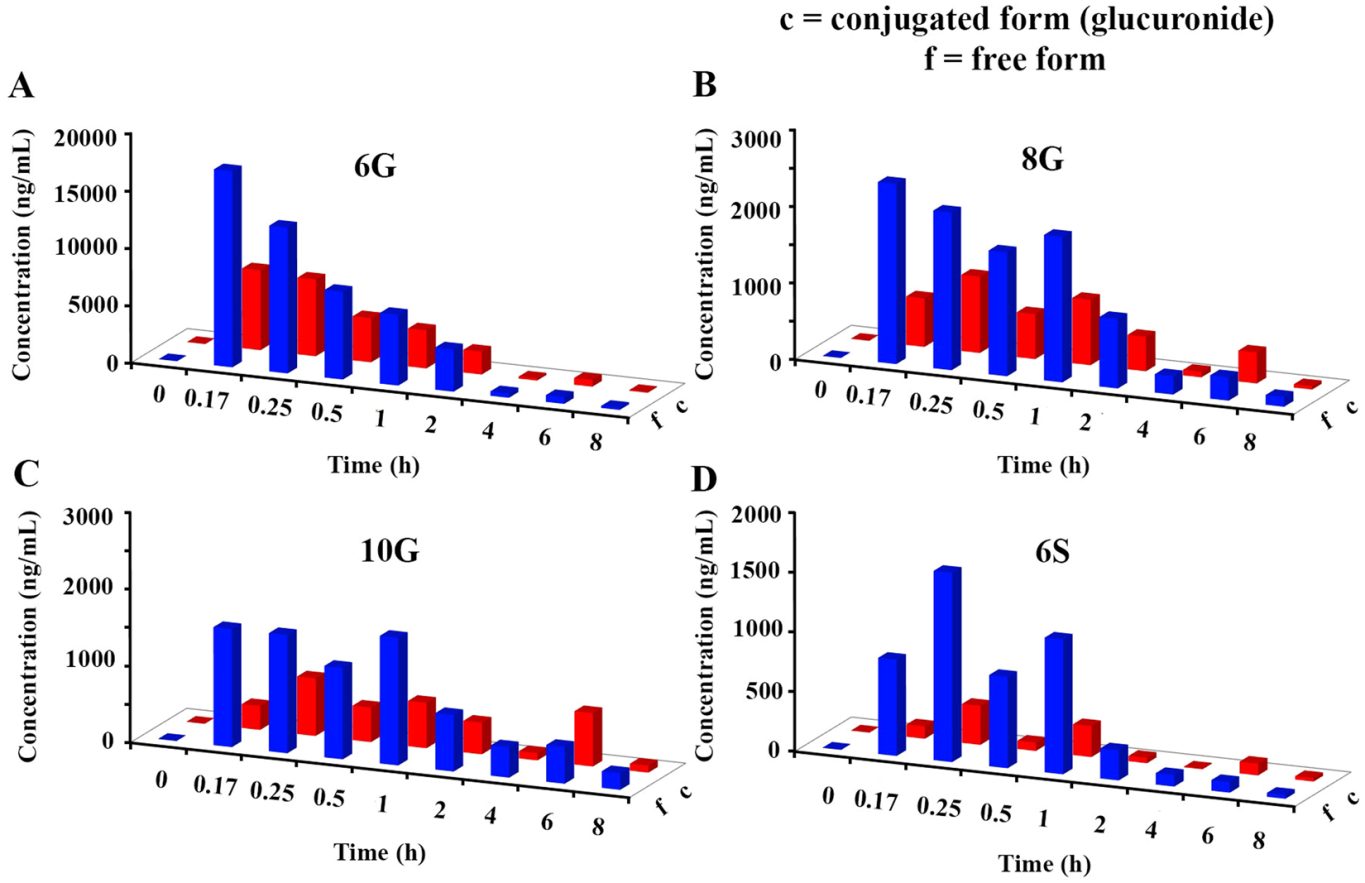
Distribution of GPs in the stomach following repeated dose administration of GE. Concentrations of GPs in untreated and β-gd treated samples obtained from stomach were compared at different time points (n = 3). Three-dimensional representation of graphs in Microsoft Excel does not permit SD inclusion and hence, the SD values are provided in Supplementary Table 1.

The sum of exposure (AUC_last_) of free 6G, 8G, and 6S, in various organs was more than that in the plasma suggesting extensive tissue distribution. Conversely, the AUC_last_ of free 10G was higher in plasma compared to all other tissues (Supplementary Table 10). The ratio of conjugated to free AUC_last_ suggested a higher accumulation of conjugated GPs in heart (10.22–18.54) and lung (2.50–5.57). In all other tissues, the values of conjugated to free forms ranged from 0.2 to 2 (Supplementary Table 11) except for brain (0.19–10.25) and tumor (9.67 for 6S). Notably, conjugates of GPs were present in the brain samples (Fig. 2), suggesting that the conjugated forms are able to cross the blood-brain barrier. Further, normalization of total exposure (AUC_last_ of free and conjugated form) to organ weight (Supplementary Table 12) revealed that the accumulation of 6G, 8G and 10G was higher in kidney compared to other organs except stomach. The accumulation of 6G was 2–5 fold higher in lung, spleen, and liver (Supplementary Table 13) compared to other GPs. Another interesting observation in this study was the presence of glucuronide conjugates of GPs in the stomach of the mouse (Fig. 3).

Given that mouse stomach is known to express UGTs and its pH is less than 4.0 under fed or fasted conditions, the presence of conjugated forms at this low pH confirm their formation and stability at such acidic pH. In addition, the concentration of GPs and their conjugates were significantly lower in the intestine compared to stomach suggesting their rapid absorption from the intestine.

### β-glucuronidase over-expression and tumor accumulation of GPs

**Figure 4:**
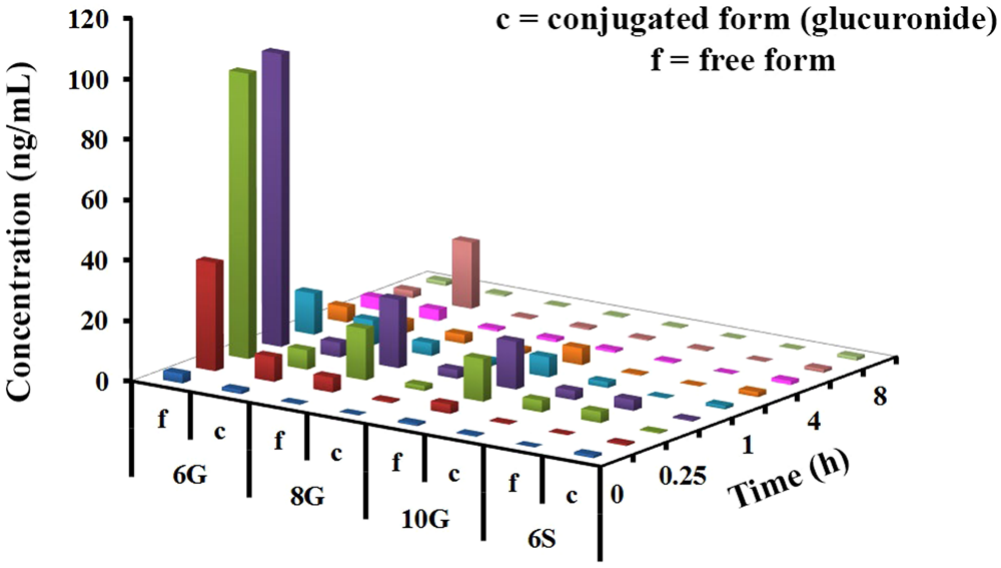
Tumor concentration of GPs following repeated dose administration of GE. Concentrations of GPs in untreated and β-gd treated tumor samples were compared at different time points (n = 3). Three-dimensional representation of graphs in Microsoft Excel does not permit SD inclusion and hence, the SD values are provided in Supplementary Table 9.

β-glucuronidase (β-gd) is overexpressed in most cancer cells with high variability reported in its expression in patients. In human prostate cancer, its expression was 3.6-fold higher than normal tissue (57.5 ± 33.1 versus 16.9 ± 8.5, μg of phenolphthalein liberated/h, activity per 10 mg of tissue)^19^. In healthy human pancreatic tissues, the level of β-gd was lower (median: 74) compared to cancerous tissue (median 133) measured using liberation of 4-Methylumbelliferyl-β-D-glucuronide^20^. Similarly, in bladder cancer patients the values of β-gd were significantly different (mean ± S.E. of β-gd/mg creatinine; normal (n = 125, 57.3 ± 2.2); patients in remission (n = 15, 69.6 ± 5.7); patients with disease (n = 6, 135.0 ± 21.8))^21^. Further, it is also reported that dosing rats bearing colon cancer with GE (50 mg/kg, QD, oral for 30 weeks), reduced the activity of β-gd^22^. Our *in silico* analysis showed statistically significant over-expression of β-gd in stomach, intestine, lung, brain, liver, kidney, prostate, pancreatic and breast cancer tissues compared to normal ones (Supplementary Figure 2). Thus, our next step was to confirm the presence of free forms and glucuronide conjugates of GPs in the tumor. To do so, we first analyzed tissue homogenates as such. To a separate aliquot, β-gd was added and the mixture was incubated for 1 h at 37 °C to release the conjugated GPs. This experiment led to an increase in concentration of free GPs confirming the presence of conjugated GPs in tissues. The analysis of tumor samples collected on day 7 revealed that the accumulation of 6G was the highest among all the GPs (Table 2, Fig. 4, Supplementary Table 9), although the plasma exposure of 10G was the highest (Supplementary Table 10).

**Table 2:** Pharmacokinetic parameters of GE phenolics in the tumor isolated on day 7 following daily oral administration of GE in nude mice (dose: 250 mg/kg, n = 3 per time point, mean ± SE).

Analyte	Free/Conjugate	Pharmacokinetic Parameters		
		T_max_ (h)	C_max_ (ng/mL)	AUC_last_ (ng.h/mL)
6G	F	0.5	96.79 ± 3.24	93.66 ± 4.75
	C	1.0	8.24 ± 1.11	29.00 ± 4.41
	Ratio c/f	—	0.085	0.31
8G	F	0.5	22.58 ± 1.42	22.33 ± 0.83
	C	0.5	2.92 ± 1.71	10.25 ± 2.98
	Ratio c/f	—	0.13	0.46
10G	F	0.5	15.78 ± 0.94	25.87 ± 2.63
	C	0.25	3.88 ± 0.93	5.68 ± 0.93
	Ratio c/f	—	0.25	0.22
6S	F	0.25	3.67 ± 0.30	2.89 ± 0.18
	C	2.0	1.63 ± 0.20	27.66 ± 8.72
	Ratio c/f	—	0.44	9.57

T_max_: time to reach peak plasma concentration; C_max_: peak plasma concentration; AUC_last_: area under the curve; SE: standard error; f: free form; c: conjugated form (glucuronide).

Interestingly, accumulation of conjugated 6S was 9-fold higher compared to its free form in the tumor, while in the case of 6G, 8G, and 10G, the conjugates exposure was about 20–50% lower compared to the free forms. The exposure (free + conjugated form) of 6G, 8G, 10G and 6S was 123, 33, 32 and 31 ng·h/mL (Supplementary Table 13), respectively, suggesting a total peak concentration of 200 ng·h/mL in tumor tissue.

### *In vitro* cytotoxicity of free GPs as well as the conjugated form

We next wanted to assess whether free GPs or the conjugated forms are responsible for efficacy. We performed *in-vitro* cell proliferation assays using PC-3 and DU145 prostate cancer cells. We found no cytotoxicity of 6G-phenolic glucuronide on PC-3 or DU145 prostate cancer cells up to the highest concentration tested (200 µM). All GPs showed IC_50_ values ranging from 30 µM for 10G to 196 µM for 6G (Table 3), confirming that free forms are the active components responsible for GPs anticancer efficacy and not the conjugated forms.

**Table 3:** Cytotoxicity (IC_50_ values) of GE phenolics and 6G-glucuronide conjugate in PC-3 and DU145 cancer cells.

Cells	IC_50_ (µM, n = 4, mean)				
	6-gingerol	8-gingerol	10-gingerol	6-shogaol	6G glucuronide
PC-3	160	108	30	102	> 200
DU145	196	182	31	37	> 200

Doxorubicin and blank media samples were run as controls.

## Discussion

Although NMs like whole ginger extract have been in the spotlight as complementary and alternative medicines, the sub-therapeutic concentrations of their active phenolics achieved *in vivo* have slowed their clinical development^16^. The primary reason for this setback is the prevalent belief that the glucuronide and sulfate conjugates do not show any pharmacological activity^23,24^. In addition, the tissue distribution of these components has not been studied. Efforts have also been diverted to developing the nanosuspensions or PEGylated NMs to increase their oral bioavailability^25–28^. The current investigation highlights the importance of monitoring the right analytes/metabolites for studying NMs.

Ginger has been shown to exert a variety of therapeutic and preventive effects for several ailments ranging from common cold to cancer^4,13,29–33^. Since the concentration of GPs is high in fresh ginger, whereas shogaols are abundant in the dried form^34–36^, we considered gingerols and shogaol as markers for the fresh and the dried conditions, respectively. It is also known that temperature and pH convert gingerols to shogaols and *vice versa*^37^. Our earlier study demonstrating enterohepatic recirculation of GPs and the formation of their glucuronide conjugates^15^ piqued our interest, and we set out to investigate the tissue distribution of active GPs, 6G, 8G, 10G and 6S and their glucuronide conjugates to gain insights into the basis of ginger’s anticancer activity. To further understand the role of conjugates *in vivo*, we quantitated the conjugated forms relative to their free forms in a various tissues including tumor. The distribution of GPs revealed that their conjugated forms accumulate in addition to the free forms within various tissues and tumor (Figs 2 and 3). Their presence within the kidneys (Fig. 2) can be explained based on the typical water solubility of conjugates to favor successful elimination from the system. To the best of our knowledge, we are the first to report the accumulation of free and conjugated GPs in various organs including the tumor and brain (Fig. 2). Our data suggest that GPs conjugates can cross the blood-brain barrier (BBB) to accumulate in the brain. Considering that plasma volume (3 mL) is 100-fold higher than cerebrospinal fluid (30 µL) in a mice of 30 g body weight, the concentrations seen in brain are similar to that seen in plasma and therapeutically significant^38^.

Our tumor distribution data showed the predominance of conjugated form of 6S than its free form (Supplementary Table 11). The exposure ratio (AUC_last_) of the 6S conjugate in tumors was 9-fold higher compared to the free form, while 6G, 8G, 10G displayed higher concentration of free form. Notably, both 10G and 6S showed highest anticancer efficacy *in vitro* among tested GPs. Thus, our results reveal an interesting scenario where the most cytotoxic GPs accumulate in tumors as both free and conjugated forms. From the T_max_ and C_max_ values, it is apparent that 6G, 8G, and 10G accumulate in the tumors first. This is supported by the fact that the peak levels of 6G, 8G, and 10G free forms occur at 10–15 min. Given the concentration and the length of time, these conjugates are converted to free forms in the tumor as 6G, 8G, and 10G peaked up first, followed by 6S (Table 2, Fig. 4).

β-glucuronidase, an enzyme present in cells, cerebrospinal fluid, and gastrointestinal tract, is known for its deconjugation potential of glucuronide conjugates^39^. It is widely reported that cancer cells have higher expression of β-gd compared to normal cells and increase proliferation, invasivity, metastatic spread and immune-paresis due to its deconjugation potential of endogenous glucuronide conjugates^40–42^. The analysis of *in silico* data showed statistically significant differences in β-gd levels in healthy and cancerous tissues. It is likely that these GPs conjugates take advantage of enhanced β-gd expression in cancer cells to deconjugate themselves at a faster rate compared to normal cells thus offering more specificity for cancer cells compared to healthy cells. Unlike synthetic drugs, it is intriguing how Mother Nature endowed natural phenolics with tumor-specific selectivity. In day 7 pre-dose samples, all GPs showed higher conjugate concentrations compared to free GPs in all tissues reconfirming the importance of conjugates. MTT cytotoxicity assay clearly showed the superior antiproliferative activity of GPs compared to 6G-glucuronide.

**Figure 5:**
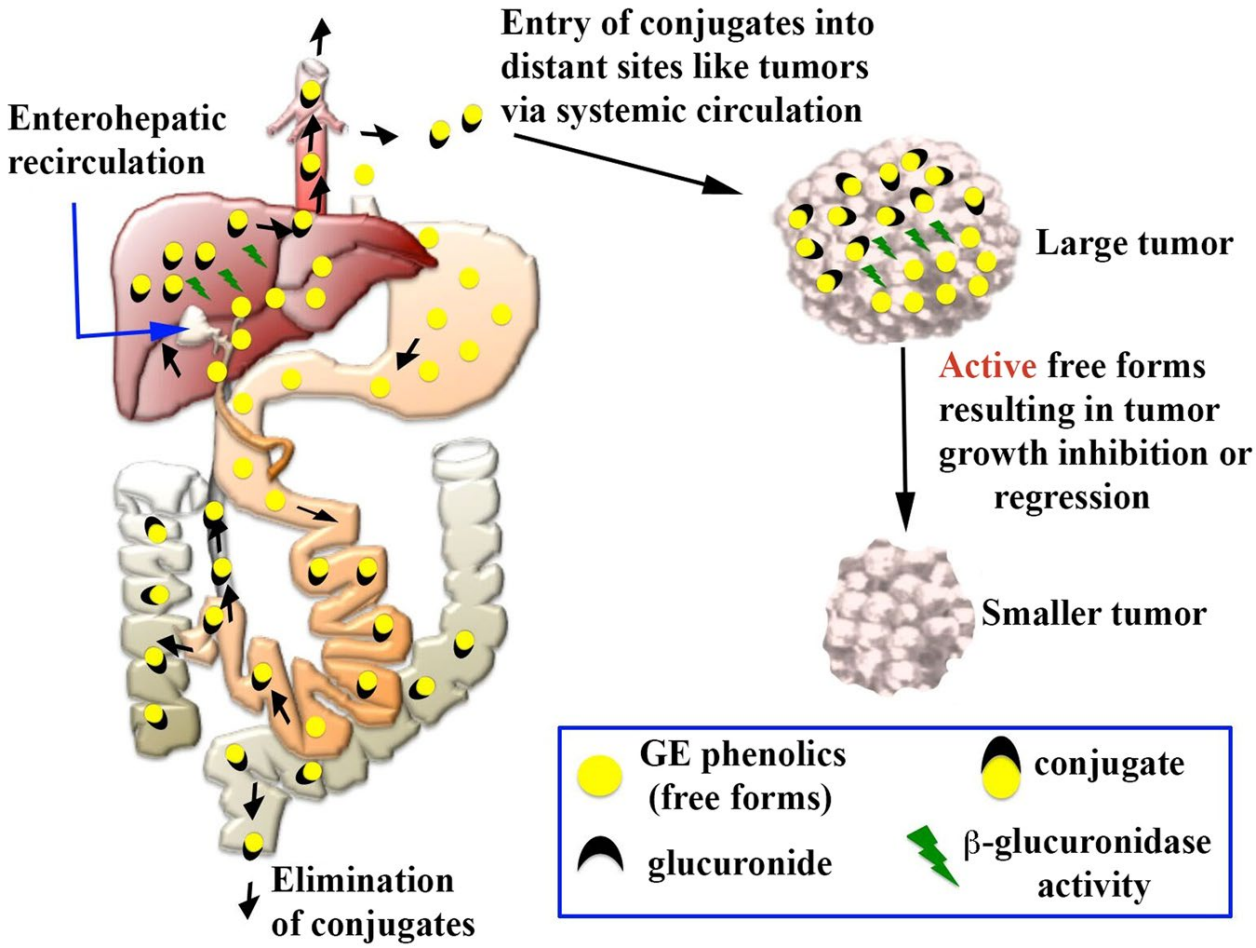
Proposed mechanism of action of GPs. After oral administration of GE, the free GPs form glucuronide conjugates in stomach, intestine, and liver. These conjugates enter the systemic circulation and get accumulated in various tissues. Once in the tissues, the conjugates are deconjugated to release the free active forms via the action of the β-glucuronidase enzyme that is expressed in high amounts in the tumor cells compared to normal cells, thus showing pharmacological effect.

The development of NMs has not garnered enough interest and attention perhaps due to the limited bioavailability of whole food components and the lack of understanding of the mechanism of action^26^. We believe that the constituent phenolics of whole foods have an inherent capacity or natural propensity to form phase II metabolites, which enter the blood stream, distribute extensively due to water solubility and accumulate in various body organs, thus contributing towards the tumor growth inhibitory efficacy by releasing free phenolics once they accumulate in the tumor cells. Considering our current observations, we propose a possible mechanism (illustrated in Fig. 5), which nullify the issues of low bioavailability by forming water soluble and permeable glucuronide conjugates which accumulate in tumor tissue and release free phenolics which in turn are responsible for efficacy.

In addition, the US FDA drug-drug interaction guidance document (2012) necessitates identification of the responsible UGTs if glucuronidation is responsible for greater than 25% of total metabolism. Typically UGT1A1, 1A3, 1A4, 1A6, 1A9, 2B7 and 2B15 can be assessed using recombinant UGT isozymes or inhibitors of UGTs like atazanavir for UGT1A1 and probenecid and valproic acid for others. Furthermore, the characterization of UGTs responsible for biotransformation of GPs is necessary to consider any DDIs during polypharmacy^43^.

Tumor environment is like an *unbeatable fortress*, wherein cancer cells show high expression of p-glycoprotein (Pgp) - the efflux protein^44^, βgd (angiogenesis and metastasis)^45^, high kinase activity (angiogenesis)^46^ and glutathione transferases (scavenger for free radicals)^47^. While current strategies somewhat rely on engaging a single target, we propose the notion of “*pharmacokinetic engineering”*, wherein customized combinations of various NMs, marketed drugs and new chemical entities can be tailored to effectively eradicate cancer by targeting the entire tumor landscape- tumor cells as well as the stromal milieu.

To summarize, this study examined the role of GP conjugates, the optimal PK analyte “chemical markers” such as 6G, 8G, 10G and 6S along with their conjugates, and “biomarkers” like β-gd in facilitating enhanced accumulation within target tissues to exert favorable PD effects. This strategy can be extrapolated to other NMs for bridging the PK-PD gap for rational development.

## Materials and Methods

### Cell Culture, Chemicals and Reagents

Ginger extract (GE) was a generous gift from Sabinsa Corporation, NJ, USA. We used 6G, 8G, 10G and 6S as markers and their concentrations were 30.03, 6.86, 7.77 and 5.96 μg/mg of ginger extract, respectively. The sum of all concentrations equals 5% of the whole extract, which is similar to what was employed in the clinical studies. Acetonitrile (ACN) and methanol were purchased from Fisher Scientific (Pittsburgh, PA). Luciferase-expressing PC-3 cells (PC-3-luc) were from PerkinElmer (Hopkinton, MA) and were maintained in modified Eagle’s medium with 10% fetal bovine serum obtained from Hyclone (Pittsburgh, PA). The PC-3-luc cells were immediately expanded and frozen down for future use (every 3 months from a frozen vial) of the same batch of cells. We used the Universal Mycoplasma Detection Kit from ATCC (ATCC, Cat#30–1012 K, Manassas, VA) to ensure that all the cell lines used were devoid of mycoplasma contamination. Carboxyl methylcellulose (CMC), nicotinamide adenine dinucleotide phosphate (NADPH), and β-glucuronidase were from Sigma (St Louis, USA). 6-gingerol glucuronide standard was procured from Santa Cruz Biotechnology (Dallas, USA). The active ginger constituents, 6G, 8G, 10G and 6S, were isolated from GE by column chromatography and were characterized for >98% purity by HPLC (High-performance liquid chromatography). All other reagents used in the study were of analytical grade.

### Pharmacokinetic studies of GPs on day 1 vs. day 7

All animal experiments were done with approval from Georgia State University’s Institutional Animal Care and Use Committee (IACUC) guidelines and under animal research protocol No. A14031. All guidelines were strictly adhered to while performing animal experiments. Pharmacokinetic studies were performed in male C57BL6J (8–10 weeks old, 25–30 g body weight, Harlan Laboratories, Indianapolis, IN) mice following oral (PO) administration of GE at 250 mg/kg (suspended in 0.5% sodium CMC) for seven days, where blood samples were collected on day 1 and day 7 post dosing. All animals were acclimatized for 5 days before dosing in the experimental area. Food and water were provided *ad libitum* throughout the study period. Animals were marked and housed (three per cage, n = 12 per group) in polypropylene cages and maintained in controlled environmental conditions with 12 h light and dark cycles. The temperature and humidity of the room were maintained between 22 ± 3 °C and 30 to 70%, respectively, and approximately 10–15 fresh air change cycles per hour. A sparse sampling design was used to collect blood samples (150 μL) from animals at 0, 0.08, 0.16, 0.25, 0.5, 1, 2, 3, 4, 6, 8 and 12 h into K_2_EDTA coated tubes. At each time point, 3 animals were used for blood sampling and from each animal, 3 blood samples were withdrawn, the last being terminal sampling by cardiac puncture. Plasma was harvested from blood by centrifugation of samples at 8000 g for 10 min. All samples were stored below −80 °C until bioanalysis.

### Pharmacokinetics and tissue distribution study in tumor-bearing mice

Six-week-old male nude C57BL6J mice (n = 27, Harlan Laboratories, Indianapolis, IN) were subcutaneously injected with PC-3-luc cells (1 × 10^6^) on the right flank. After mice (25–30 g body weight) developed palpable tumors (tumor volume of 80–100 mm^3^), were fed with 250 mg/kg GE (suspended in 0.5% sodium CMC, allometrically scaled from human dose) every day by oral gavage daily for 7 days. The dose volume used was 5 mL/kg. On Day 7, terminal blood was collected through cardiac puncture and tissue samples (stomach, intestine, brain, heart, liver, spleen, lung, kidney and tumor) were collected at 0, 0.8, 0.25, 0.5, 1, 2, 4, 6, and 8 h by euthanizing the mice. Georgia State University’s Institutional Animal Care and Use Committee (IACUC) guidelines and animal research protocol (No. A14031) were strictly adhered to while performing animal experiments.

### Enzymatic hydrolysis of GP conjugates

Each plasma and tissue sample was analyzed twice. Once directly by protein precipitation using acetonitrile and the other following treatment with β-gd. While the analysis of the sample directly by protein precipitation yielded the concentration of free GPs, analysis with β-gd resulted in total GPs concentration (free GPs + glucuronide conjugates). To confirm the presence or accumulation of GP glucuronide conjugates, plasma and tissue samples (homogenized with phosphate buffer saline, 1 part of tissue: 9 parts of buffer) were treated with β-gd (20 μL, 500 units) and incubated at 37 °C for 1 h. These samples were further processed by protein precipitation before bioanalysis.

### *In silico* analysis of β-glucuronidase expression using Oncomine

The expression level of β-gd in healthy or cancerous tissues in humans was analyzed using Oncomine database (https://www.oncomine.org). Reporter ID and platform for datasets used were as follows: gene rank 1723/18823, 2688/19574, 842/12624, 2275/18823, 2609/19574, 997/19574, 941/19189, 3345/19273 and 756/18823 analyzed on human genome U133 plus 2.0 array for gastric, colon, brain, liver, pancreas, lung, prostate, breast and renal, respectively. We selected a p value of < 0.01 as a threshold for analysis.

### Cytotoxicity assay

Cytotoxicity of GPs 6-gingerol (6G), 8-gingerol (8G), 10-gingerol (10G), 6-shogaol (6S), and 6G-phenolic glucuronide conjugate were assessed in prostate (PC-3 and DU145) cancer cell lines. Cells were cultured in T-75 flasks, and 5000 cells per well were plated in a 96-well plate. The plate was incubated overnight at 37 °C/5% CO_2_ for cell adherence. After 24 h incubation, the media was aspirated and spiked with media containing individual GE phenolics like 6G, 8G, 10G, 6S 6G-glucuronide at the highest concentration of 200 µM and subsequent 7 serial dilutions (100, 50, 25, 12.5, 6.25, 3.13, 1.60 μM). After 4 h, the media was aspirated and MTT solution (0.2 mg/mL) was added and further incubated for 2 h. To each well, 100 µL of DMSO was added to lyse the cells. The cell lysates were aspirated and centrifuged. The supernatant was collected and read at 570 nm in a spectrophotometer. Doxorubicin and blank buffer samples were used as controls in this study. No test inhibitor sample was considered as 100%, to calculate percentage inhibition. Each experiment was run in duplicate.

### Bioanalysis

Tissue samples were weighed and homogenized with phosphate buffer saline (pH 7.4, 1:10 dilution i.e. 1 part of tissue was diluted with 9 parts of buffer). All plasma and tissue samples were processed by using protein precipitation method. An aliquot (20 μL) of the sample was spiked with 200 μL of acetonitrile containing dihydrocapsaicin as the internal standard and vortex mixed for 3 min. The tubes were centrifuged at 12000 *g* for 10 min, and an aliquot of 200 μL supernatant was transferred into 1.5 mL HPLC injection vials for analysis. The stock solutions of 6G, 8G, 10G, 6S and dihydrocapsaicin (internal standard) were prepared in ACN: water (95:5) at 1 mg/mL. A calibration curve range of 0.1–500 ng/mL was employed for the quantification of analytes and internal standard (IS) concentration was 50 ng/mL for each analysis. The calibration curve consisted of blank, blank with internal standard and seven non-zero calibration standards. The calibration standards were within ± 15% of the nominal concentration, and lower limit of quantification was within ± 20% of nominal. All samples were analyzed using LC-MS/MS method (Agilent 6410 series) employing calibration curve and interspersed with quality control samples. All other analytical parameters are similar as reported earlier^15^.

### Pharmacokinetic analysis

The pharmacokinetic (PK) parameters were calculated from the concentration-time data using the non-compartmental analysis tool of Phoenix software (version 6.3, Certara, USA). The area under the concentration-time curve (AUC_last_ and AUC_inf_) was calculated using the linear trapezoidal rule. Following oral administration, peak concentration (C_max_) and time for to peak concentration (T_max_) were the observed values.

### Statistical analysis

Pharmacokinetic parameters C_max_ and AUC_last_ are reported as mean ± SEM and concentration-time profiles have mean ± SD. Student’s t-test was used to compare the day 1 and day 7 exposures, and a p value of less than 0.05 was considered significant.

## Electronic supplementary material

Supplementary Information
