# Supplementary material for: Pharmacokinetic-pharmacodynamic correlations in the development of ginger extract as an anticancer agent
Source: Sci Rep. 2018 Feb 14;8:3056. doi: 10.1038/s41598-018-21125-2 (PMC5813242; doi:10.1038/s41598-018-21125-2)

Supplementary information for

Pharmacokinetic-pharmacodynamic correlations in the development of ginger extract as an anticancer agent

Rao Mukkavilli[1], Chunhua Yang[1], Reenu Singh Tanwar[1], Roopali Saxena[1], Sushma R. Gundala[1], Yingyi Zhang[1], Ahmed Ghareeb[1], Stephan D Floyd[1], Subrahmanyam Vangala[2], Wei-Wen Kuo[3], Padmashree CG Rida[1], Ritu Aneja[1]

[1]Department of Biology, Georgia State University, Atlanta, GA-30303

[2]ReaGene Biosciences Private Limited, Bengaluru, India

[3]Department of Biological Science and Technology, College of Biopharmaceutical and Food Sciences, China Medical University, Taichung, Taiwan, ROC

*Correspondence Author:

Ritu Aneja, PhD

Department of Biology, Georgia State University, Atlanta, GA 30303

Email : raneja@gsu.edu; Phone : 404-413-5417; Fax: 404-413-5301

This supplementary information includes Supplementary Tables 1 to 13 and Supplementary Figures 1 to 2.

Supplementary Table 1

Concentrations of conjugated (c) and free (f) forms of GE phenolics in stomach following daily dose administration of GE (n=3 per time point, mean ± SD)

Time (h)	Concentration (mean ± SD, ng/mL)							
	6G		8G		10G		6S	
	c	f	C	F	C	f	C	f
Pre-dose*	2.05 ± 1.58	5.02 ± 1.76	0.10 ± 0.01	0.10 ± 0.01	0.12 ± 0.10	1.15 ± 0.26	0.61 ± 0.65	1.73 ± 0.28
0.16	6956.64 ± 1217.51	17077.16 ± 482.27	632.29 ± 474.35	2349.25 ± 101.98	310.12 ± 175.98	1537.09 ± 43.69	102.48 ± 115.63	804.68 ±70.45
0.25	6674.72 ± 1898.18	12701.44 ± 2961.32	955.33 ± 705.80	2055.37 ± 640.28	749.61 ± 516.87	1538.63 ± 532.30	325.66 ± 184.48	1584.02 ± 402.94
0.5	3842.96 ± 1231.79	7632.29 ± 368.48	585.43 ± 351.22	1614.75 ± 185.02	448.69 ± 299.93	1190.64 ± 137.61	68.69 ± 28.18	763.68 ± 60.66
1	3307.34 ± 1076.82	6155.38 ± 928.22	850.74 ± 651.20	1897.19 ± 497.96	589.80 ± 383.76	1661.25 ± 420.19	258.17 ± 176.65	1127.20 ± 191.35
2	1976.02 ± 505.05	3677.81 ± 403.48	448.42 ± 347.11	906.03 ± 185.47	410.59 ± 286.52	734.32 ± 138.30	43.70 ± 49.25	251.69 ± 38.82
4	172.36 ± 21.50	389.25 ± 48.80	71.09 ± 29.79	239.25 ± 67.22	94.18 ± 52.53	394.26 ± 107.11	5.50 ± 0.51	96.00 ± 23.62
6	577.59 ± 169.62	585.49 ± 32.89	402.63 ± 144.65	304.68 ± 34.41	693.61 ± 195.00	478.68 44.00	97.54 ± 32.57	88.52 ± 9.53
8	58.93 ± 27.74	181.23 ± 27.02	42.65 ± 42.88	126.79 ± 48.54	89.86 ± 95.13	216.35 ± 88.87	28.07 ± 5.50	32.71 ± 7.04

SD: Standard deviation; LLOQ: 0.1 ng/mL; *trough levels on day 7 after 6 days GE dosing

Supplementary Table 2
Concentrations of conjugated (c) and free (f) forms of GE phenolics in intestine following daily dose administration of GE (n=3 per time point, mean ± SD)

**Time (h)**	**Concentration (mean ± SD, ng/mL)**							
	**6G**		**8G**		**10G**		**6S**	
	**c**	**f**	**C**	**F**	**C**	**f**	**C**	**f**
Pre-dose*	2.54 ± 0.53	4.43 ± 1.14	0.4 ± 0.35	1.46 ± 0.36	0.26 ± 0.15	1.66 ± 0.16	1.91 ± 0.54	0.40 ± 0.52
0.16	89.59 ± 19.44	156.77 ± 10.66	16.37 ± 1.41	26.41 ± 1.37	16.00 ± 0.99	24.77 ± 0.88	1.02 ± 0.28	13.74 ± 1.01
0.25	164.79 ± 9.74	362.29 ± 17.91	18.42 ± 1.04	37.30 ± 1.11	29.63 ± 0.39	45.10 ± 1.69	0.59 ± 0.50	16.28 ± 0.30
0.5	33.58 ± 19.48	220.90 ± 19.10	2.59 ± 1.61	28.00 ± 0.62	3.63 ± 1.64	38.72 ± 1.39	6.03 ± 1.92	4.96 ± 0.22
1	32.09 ± 6.01	315.94 ± 13.16	20.40 ± 5.67	85.09 ± 1.99	23.96 ± 5.38	126.66 ± 2.18	0.72 ± 0.53	26.90 ± 2.11
2	41.28 ± 7.13	180.42 ± 15.37	14.72 ± 5.24	48.33 ± 3.29	13.88 ± 6.08	54.20 ± 4.36	1.03 ± 0.53	10.47 ± 0.61
4	28.12 ± 2.39	61.24 ± 2.51	3.40 ± 1.70	26.99 ± 1.38	11.91 ± 2.45	45.72 ± 2.88	3.74 ± 0.23	5.10 ± 0.26
6	12.48 ± 2.39	51.65 ± 2.01	0.70 ± 0.43	24.58 ± 0.09	4.60 ± 2.13	38.66 ± 1.65	2.80 ± 1.38	4.22 ± 0.14
8	5.75 ± 1.87	27.19 ± 1.19	1.48 ± 0.52	11.05 ± 0.74	3.57 ± 1.33	14.63 ± 0.47	0.75 ± 0.26	2.07 ± 0.42

SD: Standard deviation; LLOQ: 0.1 ng/mL; *trough levels on day 7 after 6 days GE dosing

Supplementary Table 3
Concentrations of conjugated (c) and free (f) forms of GE phenolics in liver following daily dose administration of GE (n=3 per time point, mean ± SD)

**Time (h)**	**Concentration (mean ± SD, ng/mL)**							
	**6G**		**8G**		**10G**		**6S**	
	**c**	**F**	**C**	**F**	**C**	**f**	**C**	**f**
Pre-dose*	5.38 ± 1.73	0.15 ± 0.06	0.10 ± 0.07	0.10 ± 0.01	0.00	0.10 ± 0.01	1.62 ± 0.19	0.00
0.16	100.05 ± 39.63	641.57 ± 42.62	3.31 ± 3.31	55.43 ± 3.12	6.98 ± 2.85	65.31 ± 0.35	1.52 ± 0.67	11.68 ± 0.12
0.25	21.50 ± 14.62	458.05 ± 15.61	3.54 ± 2.76	36.86 ± 1.53	8.58 ± 4.62	68.07 ± 7.34	1.36 ± 0.74	11.62 ± 0.80
0.5	20.62 ± 10.13	286.74 ± 9.19	4.08 ± 3.01	50.04 ± 1.25	16.23 ± 1.80	80.92 ± 5.57	1.18 ± 0.86	9.19 ± 0.21
1	27.50 ± 1.96	57.19 ± 3.34	3.16 ± 0.36	13.54 ± 0.99	20.93 ± 1.27	24.74 ± 1.73	0.97 ± 0.26	3.36 ± 0.08
2	7.62 ± 1.65	33.87 ± 0.52	1.42 ± 0.73	7.09 ± 0.25	1.57 ± 0.46	13.57 ± 0.85	0.91 ± 0.33	1.25 ± 0.20
4	37.04 ± 3.37	30.60 ± 0.93	8.98 ± 1.28	8.68 ± 0.74	20.39 ± 1.25	13.32 ± 0.55	1.28 ± 0.66	4.10 ± 0.56
6	4.58 ± 1.71	11.14 ± 0.48	1.80 ± 0.16	4.01 ± 0.05	9.98 ± 0.59	5.32 ± 0.55	1.05 ± 0.49	1.64 ± 0.10
8	1.27 ± 0.48	5.97 ± 0.10	0.92 ± 0.51	2.97 ± 0.19	3.67 ± 1.14	5.74 ± 0.20	0.80 ± 0.20	0.66 ± 0.16

SD: Standard deviation; LLOQ: 0.1 ng/mL; *trough levels on day 7 after 6 days GE dosing

Supplementary Table 4
Concentrations of conjugated (c) and free (f) forms of GE phenolics in spleen following daily dose administration of GE (n=3 per time point, mean ± SD)

Time (h)	Concentration (mean ± SD, ng/mL)							
	6G		8G		10G		6S	
	c	F	C	F	C	f	C	f
Pre-dose*	0.34 ± 0.16	1.23 ± 0.20	0.00	0.18 ± 0.07	0.00	0.17 ± 0.02	1.43 ± 0.21	0.10 ± 0.04
0.16	95.40 ± 54.66	380.92 ± 51.82	2.15 ± 1.04	14.50 ± 0.92	0.35 ± 0.34	3.17 ± 0.08	2.87 ± 0.40	4.37 ± 0.05
0.25	4.74 ± 0.91	148.06 ± 6.28	1.24 ± 1.23	8.04 ± 0.37	0.68 ± 0.36	4.39 ± 0.35	0.55 ± 0.14	2.47 ± 0.41
0.5	28.17 ± 21.92	200.57 ± 3.86	2.28 ± 1.98	16.07 ± 0.30	3.60 ± 1.09	9.78 ± 0.44	0.97 ± 0.35	3.72 ± 0.13
1	5.86 ± 2.36	97.93 ± 7.14	1.47 ± 0.52	10.62 ± 1.27	1.75 ± 0.07	4.30 ± 0.60	0.71 ± 0.09	0.96 ± 0.10
2	7.33 ± 3.05	24.00 ± 2.51	0.49 ± 0.27	1.68 ± 0.24	0.68 ± 0.42	1.13 ± 0.16	1.76 ± 0.63	0.23 ± 0.01
4	7.65 ± 0.99	11.10 ± 1.37	1.52 ± 0.31	1.75 ± 0.29	1.03 ± 0.19	1.86 ± 0.20	0.57 ± 0.15	0.91 ± 0.10
6	9.12 ± 9.86	11.65 ± 0.73	2.16 ± 2.57	2.33 ± 0.22	2.77 ± 3.11	1.93 ± 0.19	2.43 ± 1.41	0.10 ± 0.02
8	2.20 ± 1.44	4.24 ± 1.44	0.20 ± 0.16	0.26 ± 0.08	0.70 ± 0.29	0.88 ± 0.71	0.84 ± 0.25	0.22 ± 0.01

SD: Standard deviation; LLOQ: 0.1 ng/mL; *trough levels on day 7 after 6 days GE dosing

Supplementary Table 5
Concentrations of conjugated (c) and free (f) forms of GE phenolics in heart following daily dose administration of GE (n=3 per time point, mean ± SD)

Time (h)	Concentration (mean ± SD, ng/mL)							
	6G		8G		10G		6S	
	c	F	C	F	C	f	C	f
Pre-dose*	1.09 ± 0.90	0.65 ± 0.27	0.15 ± 0.11	0.10 ± 0.05	0.14 ± 0.02	0.10 ± 0.02	0.85 ± 0.13	0.00
0.16	154.13 ± 44.16	9.69 ± 2.37	11.05 ± 0.75	0.15 ± 0.06	5.52 ± 0.63	0.25 ± 0.11	1.58 ± 0.27	0.00
0.25	91.20 ± 22.62	6.67 ± 1.82	9.91 ± 1.45	0.10 ± 0.01	4.75 ± 1.41	0.25 ± 0.01	1.21 ± 0.20	0.62 ± 0.14
0.5	115.89 ± 2.15	5.99 ± 0.38	17.45 ± 0.20	0.11 ± 0.02	8.74 ± 0.61	0.38 ± 0.01	3.30 ± 0.38	0.13 ± 0.02
1	24.55 ± 2.44	2.78 ± 0.32	4.45 ± 0.10	0.12± 0.10	2.68 ± 0.35	0.28 ± 0.04	0.78 ± 0.39	0.15 ± 0.07
2	13.23 ± 1.14	1.95 ± 0.14	1.33 ± 0.30	0.33± 0.03	0.74 ± 0.16	0.28 ± 0.08	1.73 ± 0.25	0.10 ± 0.01
4	15.65 ± 1.14	0.79 ± 0.09	2.26 ± 0.30	0.12 ± 0.03	1.07 ± 0.06	0.10 ± 0.02	1.22 ± 0.09	0.10 ± 0.03
6	8.60 ± 1.14	0.66 ± 0.26	1.77 ± 0.65	0.12 ± 0.05	0.87 ± 0.07	0.00	1.30 ± 0.19	0.00
8	1.01 ± 0.58	0.68 ± 0.06	0.17 ± 0.03	0.10 ± 0.01	0.00	0.10 ± 0.03	1.15 ± 0.01	0.10 ± 0.02

SD: Standard deviation; LLOQ: 0.1 ng/mL; *trough levels on day 7 after 6 days GE dosing

Supplementary Table 6
Concentrations of conjugated (c) and free (f) forms of GE phenolics in brain following daily dose administration of GE (n=3 per time point, mean ± SD)

Time (h)	Concentration (mean ± SD, ng/mL)							
	6G		8G		10G		6S	
	c	F	C	F	C	f	C	f
Pre-dose*	0.42± 0.23	2.92 ± 0.31	0.10 ± 0.04	0.17 ± 0.03	0.11 ± 0.11	0.77 ± 0.03	0.90 ± 0.03	0.10 ± 0.02
0.16	1.47 ± 1.00	12.09 ± 0.44	0.73 ± 0.22	0.79 ± 0.07	0.10 ± 0.06	0.80 ± 0.06	0.64 ± 0.23	0.10 ± 0.00
0.25	8.67 ± 4.86	6.88 ± 0.61	0.38 ± 0.24	0.32 ± 0.10	0.10 ± 0.07	0.83 ± 0.06	0.11 ± 0.06	0.10 ± 0.00
0.5	17.01 ± 1.95	4.72 ± 0.10	2.48 ± 0.50	0.20 ± 0.06	0.47 ± 0.22	1.01 ± 0.15	0.98 ± 0.16	0.10 ± 0.02
1	5.75 ± 2.23	2.49 ± 0.58	1.25 ± 0.23	0.17 ± 0.02	0.28 ± 0.10	0.96 ± 0.12	1.38 ± 0.25	0.30 ± 0.07
2	1.68 ± 0.40	0.85 ± 0.22	0.20 ± 0.10	0.10 ± 0.03	0.10 ± 0.06	0.49 ± 0.10	1.35 ± 0.40	0.10 ± 0.01
4	1.88 ± 0.41	1.16 ± 0.31	0.24 ± 0.03	0.20 ± 0.04	0.10 ± 0.04	0.61 ± 0.03	1.49 ± 0.01	0.00
6	0.41 ± 0.17	1.64 ± 0.13	0.20 ± 0.21	0.62 ± 0.21	0.13 ± 0.04	0.87 ± 0.12	1.18 ± 0.25	0.10 ± 0.01
8	1.73 ± 0.86	4.76 ± 2.34	3.00 ± 3.85	4.85 ± 3.75	0.42 ± 0.28	2.63 ± 0.29	0.80 ± 0.16	0.30 ± 0.05

SD: Standard deviation; LLOQ: 0.1 ng/mL; *trough levels on day 7 after 6 days GE dosing

Supplementary Table 7
Concentrations of conjugated (c) and free (f) forms of GE phenolics in lung following daily dose administration of GE (n=3 per time point, mean ± SD)

Time (h)	Concentration (mean ± SD, ng/mL)							
	6G		8G		10G		6S	
	c	F	C	F	C	f	C	f
Pre-dose*	0.48 ± 0.29	1.40 ± 0.13	0.10 ± 0.06	0.10 ± 0.01	0.10 ± 0.02	0.17 ± 0.02	1.20 ± 0.35	0.00
0.16	85.58 ± 16.05	98.88 ± 7.14	7.25 ± 2.73	8.22 ± 0.11	4.21 ± 1.37	3.45 ± 0.07	0.61 ± 0.23	1.44 ± 0.02
0.25	178.43 ± 6.23	104.56 ± 1.36	17.18 ± 1.04	15.28 ± 0.17	11.50 ± 0.34	8.63 ± 0.37	2.03 ± 0.60	0.63 ± 0.15
0.5	258.48 ± 16.15	75.12 ± 1.98	42.99 ± 3.37	15.20 ± 0.80	26.68 ± 3.73	10.01 ± 0.70	2.81 ± 0.72	2.45 ± 0.44
1	62.47 ± 10.08	17.94 ± 0.95	10.98 ± 1.96	3.19 ± 0.26	10.04 ± 2.11	2.87 ± 0.34	1.09 ± 0.42	0.23 ± 0.02
2	32.75 ± 4.95	14.43 ± 0.95	3.55 ± 0.66	3.22 ± 0.28	2.10 ± 0.87	2.68 ± 0.03	1.14 ± 0.50	0.10 ± 0.01
4	27.63 ± 10.18	3.15 ± 0.21	5.83 ± 2.35	0.30 ± 0.05	4.41 ± 1.40	0.46 ± 0.05	1.27 ± 0.21	0.10 ± 0.02
6	20.24 ± 1.33	4.95 ± 0.40	5.50 ± 0.43	1.81 ± 0.19	4.72 ± 0.18	1.55 ± 0.14	1.37 ± 0.30	0.00
8	6.44 ± 1.34	5.53 ± 0.87	2.09 ± 0.95	1.52 ± 0.70	1.54 ± 1.18	1.35 ± 0.78	0.91 ± 0.12	0.12 ± 0.02

SD: Standard deviation; LLOQ: 0.1 ng/mL; *trough levels on day 7 after 6 days GE dosing

Supplementary Table 8
Concentrations of conjugated (c) and free (f) forms of GE phenolics in kidney following daily dose administration of GE (n=3 per time point, mean ± SD)

Time (h)	Concentration (mean ± SD, ng/mL)							
	6G		8G		10G		6S	
	c	f	C	F	C	f	C	f
Pre-dose*	2.47 ± 0.15	0.1 ± 0.03	0.1 ± 0.15	0.1 ± 0.02	0.1 ± 0.08	0.1 ± 0.02	1.31 ± 0.11	0.00
0.16	153.35 ± 13.26	778.27 ± 33.34	28.95 ± 6.83	76.97 ± 3.09	6.64 ± 2.15	16.34 ± 0.62	3.29 ± 1.36	19.74 ± 0.38
0.25	348.81 ± 60.24	1035.58 ± 4.80	26.37 ± 7.11	173.66 ± 2.99	22.86 ± 4.94	73.32 ± 1.59	5.62 ± 0.96	30.71 ± 0.06
0.5	167.38 ± 165.23	873.36 ± 173.97	25.08 ± 11.86	196.95 ± 45.92	24.47 ± 29.07	133.25 ± 28.67	2.60 ± 1.72	26.07 ± 4.42
1	335.97 ± 49.38	131.64 ± 8.40	73.80 ± 10.21	38.57 ± 2.53	103.19 ± 22.53	35.93 ± 1.62	3.68 ± 0.57	4.54 ± 0.06
2	99.10 ± 4.24	60.50 ± 3.93	21.03 ± 0.15	18.32 ± 0.81	15.86 ± 1.61	14.00 ± 0.86	0.57 ± 0.49	1.46 ± 0.17
4	110.10 ± 3.55	22.13 ± 1.63	35.78 ± 0.34	8.89 ± 0.91	21.47 ± 1.05	4.79 ± 0.65	0.54 ± 0.19	1.10 ± 0.29
6	69.89 ± 6.82	16.47 ± 1.79	28.32 ± 5.29	6.80 ± 1.23	26.15 ± 6.09	5.32 ± 0.88	1.20 ± 0.61	0.10 ± 0.03
8	21.37 ± 1.83	6.96 ± 0.59	5.88 ± 0.08	3.35 ± 0.13	5.49 ± 0.12	2.46 ± 0.07	0.94 ± 0.22	0.2 ± 0.04

SD: Standard deviation; LLOQ: 0.1 ng/mL; *trough levels on day 7 after 6 days GE dosing

Supplementary Table 9
Concentrations of conjugated (c) and free (f) forms of GE phenolics in tumor following daily dose administration of GE (n=3 per time point, mean ± SD)

Time (h)	Concentration (mean ± SD, ng/mL)							
	6G		8G		10G		6S	
	c	F	C	F	C	f	C	f
Pre-dose*	1.23 ± 1.37	3.11 ± 0.72	0.10 ± 0.05	0.22 ± 0.11	0.40 ± 0.50	0.74 ± 0.17	0.92 ± 0.17	0.00
0.16	8.19 ± 4.31	35.79 ± 1.43	0.59 ± 0.27	4.93 ± 0.29	0.27 ± 0.12	3.14 ± 0.19	0.59 ± 0.08	0.10 ± 0.01
0.25	6.41 ± 1.65	94.36 ± 1.36	1.67 ± 0.92	16.97 ± 0.72	3.88 ± 0.93	14.05 ± 0.50	0.38 ± 0.12	3.67 ± 0.30
0.5	4.82 ± 3.78	96.79 ± 3.24	2.92 ± 1.71	22.58 ± 1.42	3.08 ± 1.36	15.78 ± 0.94	0.49 ± 0.31	3.61 ± 0.35
1	8.24 ± 1.11	13.58 ± 3.17	2.04 ± 0.69	4.16 ± 0.78	1.88 ± 1.00	6.62 ± 1.03	1.11 ± 0.15	0.17 ± 0.04
2	3.90 ± 1.90	5.04 ± 0.70	1.64 ± 1.17	2.95 ± 0.39	0.38 ± 0.34	5.55 ± 0.99	1.63 ± 0.20	0.10 ± 0.02
4	3.63 ± 0.74	4.00 ± 0.08	1.00 ± 0.68	0.77 ± 0.02	0.43 ± 0.15	0.83 ± 0.14	1.22 ± 0.10	0.00
6	2.19 ± 0.36	2.33 ± 0.24	0.63 ± 0.15	0.33 ± 0.10	0.40 ± 0.14	0.44 ± 0.16	1.10 ± 0.14	0.10 ± 0.01
8	0.50 ± 0.55	1.37 ± 0.30	0.30 ± 0.24	0.20 ± 0.05	0.10 ± 0.01	0.28 ± 0.06	1.12 ± 0.33	0.10 ± 0.02

SD: Standard deviation; LLOQ: 0.1 ng/mL; *trough levels on day 7 after 6 days GE dosing

Supplementary Table 10
Comparison of exposure (AUC_{last}) of free GE phenolics in plasma and collected tissues (n=3 per time point, mean values presented)

Organs	AUC_{last} (ng.h/mL)			
	6G	8G	10G	6S
Plasma	642	132	1597	52
Liver	449	81	132	23
Spleen	307	28	18	6
Heart	13	1	1	1
Brain	18	7	8	1
Lung	115	22	17	2
Kidney	875	189	135	26
Tumor	94	22	26	3

Supplementary Table 11
Ratio of conjugate to free GE phenolics AUC_{last} ratio
(n=3 per time point, mean values presented)

**Organs**	**Conjugated/Free AUC_{last} ratio**			
	**6G**	**8G**	**10G**	**6S**
Stomach	0.54	0.53	0.59	0.26
S.Intestine	0.24	0.20	0.22	0.29
Liver	0.31	0.36	0.69	0.36
Spleen	0.24	0.38	0.67	1.93
Heart	12.55	18.54	10.22	15.46
Brain	1.17	0.89	0.19	10.25
Lung	2.92	2.61	2.50	5.57
Kidney	1.06	1.26	1.60	0.41
Tumor	0.31	0.46	0.22	9.67

Supplementary Table 12

(a) Total exposure (free + glucuronide) of GE phenolics in collected tissues per mg of organ weight (n = 3 per time point, mean values presented)

Organs	AUC_{last} (ng.h/mL) / mg of organ weight			
	6G	8G	10G	6S
Stomach	221	59	61	21
S.Intestine	5.58	1.57	2.28	0.40
Liver	2.24	0.42	0.85	0.12
Spleen	4.70	0.48	0.37	0.21
Heart	1.38	0.18	0.10	0.09
Brain	0.14	0.05	0.03	0.04
Lung	4.12	0.74	0.55	0.11
Kidney	10.35	2.46	2.06	0.21
Tumor	0.99	0.26	0.26	0.25

(b) Weights of various organs (mg, n=3 at each time point)

Organs	Stomach	S. Intestine	Liver	Spleen	Heart	Brain	Lung	Kidney	Tumor
0 min	120	211	337	75	135	418	121	214	96
10 min	133	271	423	84	134	285	145	195	118
15 min	126	101	116	67	117	125	110	92	111
30 min	132	126	113	94	136	113	108	121	117
1 h	117	133	156	93	123	126	106	121	114
2 h	138	136	148	83	132	210	101	189	120
4 h	148	323	369	88	134	379	89	231	167
6 h	119	226	279	66	123	393	86	170	127
8 h	186	328	427	82	136	421	119	230	151
Average	135	206	263	81	130	274	109	174	125
SD	21	87	131	10	7	133	18	51	22
%CV	16	42	50	13	5	49	16	30	17

Supplementary Table 13
Total exposure AUC_{last} (free + conjugated form) of GE phenolics in various tissues (n=3 per time point, mean values presented)

Organs	AUC_{last} (ng.h/mL)			
	6G	8G	10G	6S
Stomach	29869	8007	8268	2868
S.Intestine	1150	324	469	82
Liver	590	111	223	32
Spleen	382	39	30	17
Heart	179	24	13	12
Brain	39	13	9	11
Lung	451	81	60	12
Kidney	1798	428	351	36
Tumor	123	33	32	31

Supplementary Figure 1
Structure of GE phenolics and 6 gingerol phenolic glucuronide

Name	Structure
6-gingerol	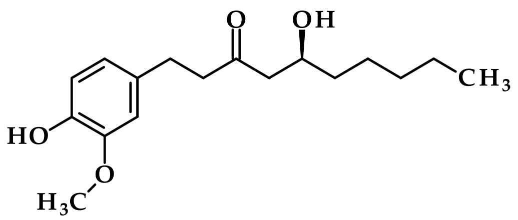
8-gingerol	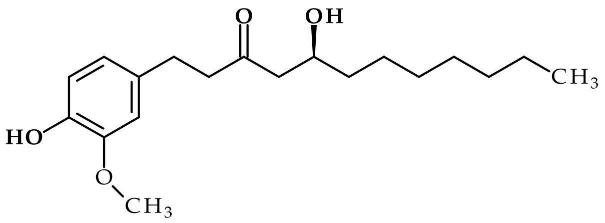
10-gingerol	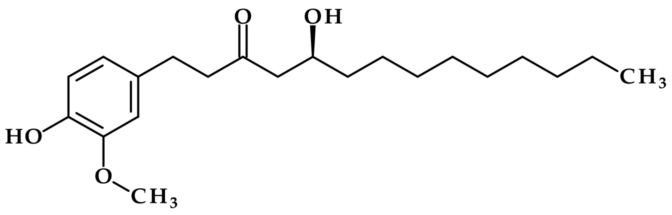
6-shogaol	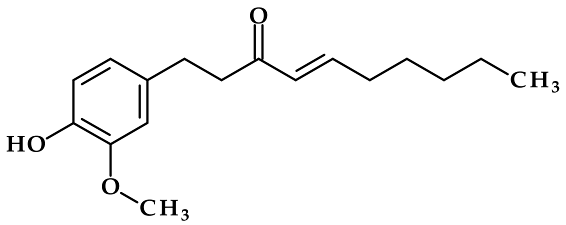
6-gingerol phenolic glucuronide	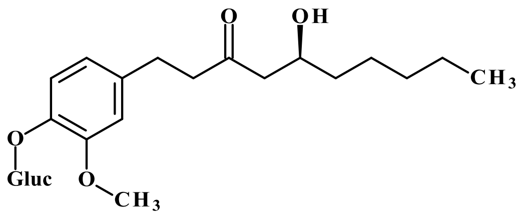

Supplementary Figure 2
In silico analysis of β-glucuronidase expression in various cancer cells

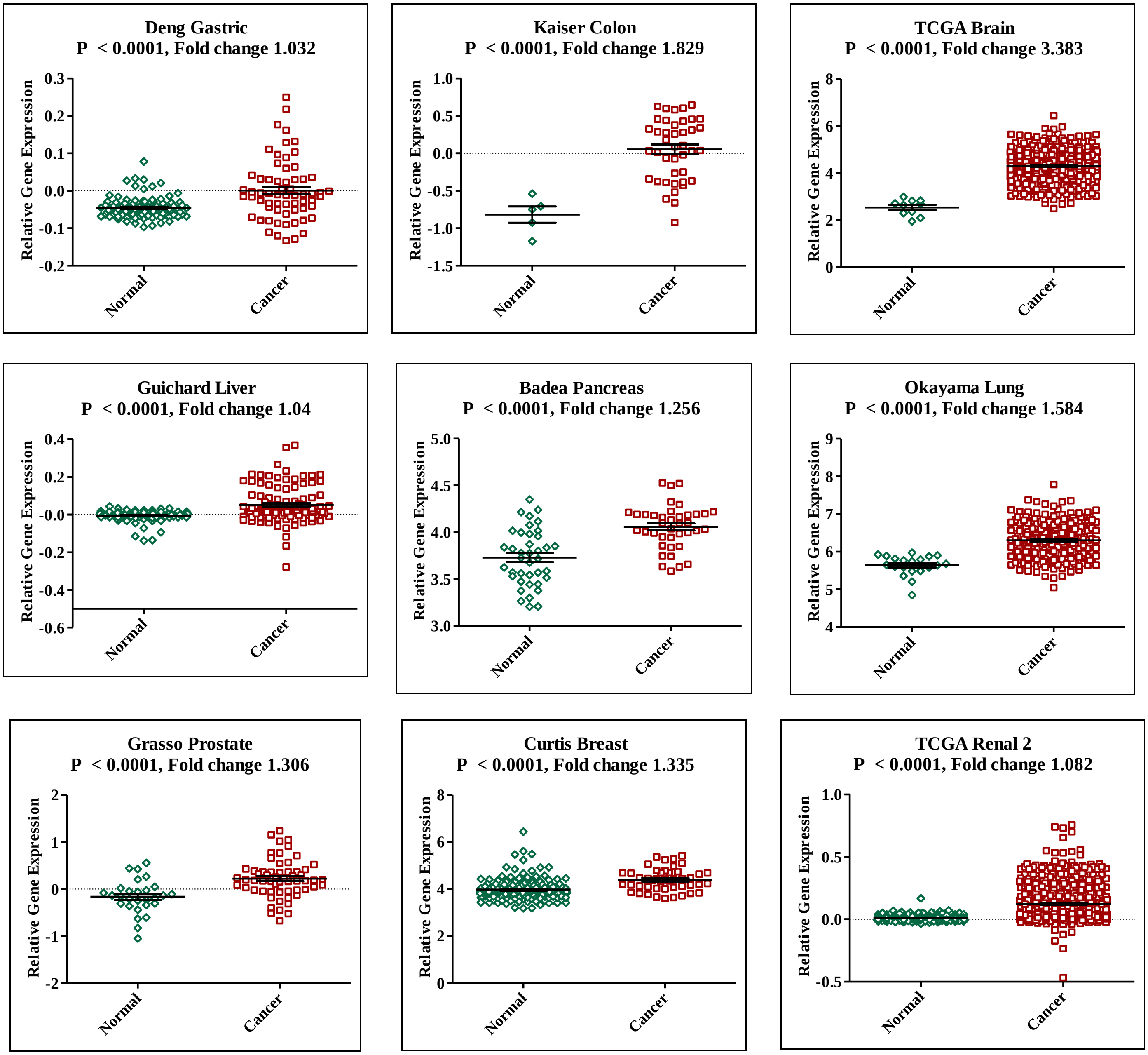

Supplement: Supplementary file 1 — Supplementary Information [file 41598_2018_21125_MOESM1_ESM.pdf]
